# Fecal Microbiota Transplantation Modulates Th17/Treg Balance via JAK/STAT Pathway in ARDS Rats

**DOI:** 10.1002/adbi.202500028

**Published:** 2025-06-27

**Authors:** Dongwei Zhang, Biying Dong, Jie Chen, Zhenqiang Zhang, Weitong Zeng, Longxiong Liao, Xia Xiong, Xuejun Qin, Xianming Fan

**Affiliations:** ^1^ Department of Respiratory and Critical Care Medicine The Affiliated Hospital of Southwest Medical University Luzhou Sichuan 646000 China; ^2^ Inflammation & Allergic Diseases Research Unit The Affiliated Hospital of Southwest Medical University Luzhou Sichuan 646000 China; ^3^ Department of Respiratory and Critical Care Medicine Liuzhou People's Hospital Guangxi Medical University Liuzhou Guangxi 545006 China; ^4^ Key Laboratory of Diagnosis Treatment and Research of Asthma and Chronic Obstructive Pulmonary Disease Liuzhou Guangxi 545006 China; ^5^ Graduate School of Southwest Medical University Luzhou Sichuan 646000 China; ^6^ Department of Dermatology The Affiliated Hospital Southwest Medical University Luzhou Sichuan 646000 China

**Keywords:** acute respiratory distress syndrome, fecal microbiota transplantation, JAK/STAT, regulatory T cells, Th17 cells

## Abstract

This study evaluated the therapeutic effects of fecal microbiota transplantation (FMT) on lipopolysaccharide (LPS)‐induced acute respiratory distress syndrome (ARDS) in rats. The study focused on the balance of T‐helper 17 (Th17) and regulatory T (Treg) cells, as well as the modulation of the JAK/STAT pathway. This study established a rat ARDS model using intranasal LPS instillation, administering interventions such as FMT, Treg cell depletion, and JAK inhibitors. Assessments included histopathological examination of lung and intestinal tissues, flow cytometry for Th17 and Treg cell proportions, qPCR and Western blot for gene and protein expression, ELISA for inflammatory cytokines, and correlation analysis using Spearman's method for cytokine‐immune cell interactions. Results indicated that FMT and JAK inhibitors significantly reduce lung damage induced by LPS, reduced alveolar destruction and inflammation, restored Th17/Treg balance, and inhibited JAK/STAT pathway activity. Notably, FMT decreased pro‐inflammatory cytokines (IL‐2, IL‐6, IL‐8, IL‐17A, IL‐23, TGF‐β1) and increased anti‐inflammatory cytokines (IL‐10, IL‐35) in serum. Spearman correlation analysis indicated that FMT restored immune balance by modulating the interactions between cytokines and immune cells. In conclusion, FMT effectively alleviates lung and intestinal injury in LPS‐induced ARDS rat models by modulating Th17/Treg balance and inhibiting JAK/STAT pathway activity, demonstrating promising therapeutic potential for ARDS treatment.

## Graphical abstract

Fecal microbiota transplantation (FMT) alleviates lung and intestinal injury in lipopolysaccharide (LPS)‐induced acute respiratory distress syndrome (ARDS) in rats. This study demonstrates that FMT restores the balance between Th17 and Treg cells, inhibits the JAK/STAT pathway, and modulates cytokine levels, reducing inflammation and lung damage. These findings suggest that FMT holds therapeutic potential for ARDS treatment by regulating immune responses and promoting tissue repair.

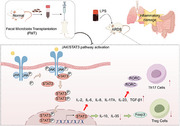

## 1 Introduction

Acute respiratory distress syndrome (ARDS) is a severe, life‐threatening, and rapidly progressive pulmonary disorder characterized by acute onset, progressive dyspnea, and hypoxemia.^[^ ^1^ ^]^ The underlying causes of ARDS are diverse, including infectious agents such as pneumonia and sepsis, as well as non‐infectious triggers such as trauma and inhalation injuries.^[^ ^2^ ^]^ Due to key pathophysiological changes, including disruption of the alveolar‐capillary barrier, infiltration of inflammatory cells, and the onset of cytokine storms, ARDS presents a high mortality rate of 30%–40%, significantly endangering patient survival.^[^ ^3^ ^]^ Current treatments for ARDS primarily focus on supportive care measures, such as mechanical ventilation, extracorporeal membrane oxygenation (ECMO), and pharmacological interventions such as glucocorticoids. However, these therapeutic options demonstrate limited efficacy in improving patient outcomes and are often associated with significant clinical complications.^[^ ^4^ ^]^ Consequently, the search for more effective therapeutic strategies to improve ARDS outcomes has become a central focus of ongoing medical research.

In recent years, growing evidence has underscored the crucial role of gut microbiota in regulating immune responses and maintaining homeostasis.^[^ ^5^ ^]^ Fecal microbiota transplantation (FMT) has emerged as a promising therapeutic approach, demonstrating potential in treating various diseases by restoring the balance of the gut microbial ecosystem.^[^ ^6^, ^7^, ^8^ ^]^ In ARDS, immune dysregulation plays a critical role in its pathogenesis, with the JAK/STAT pathway playing a key role in regulating the differentiation and function of immune cells.^[^ ^9^ ^]^ The balance between Th17 and Treg cells is essential for maintaining immune homeostasis in the lungs, and its disruption can trigger severe inflammatory responses.^[^ ^10^ ^]^ RORγt, a nuclear hormone receptor, is crucial for the differentiation of Th17 cells.^[^ ^11^ ^]^ Treg cells are characterized by the expression of Foxp3, a transcription factor that regulates their function by modulating various genes.^[^ ^12^ ^]^ The expression of these transcription factors modulates the balance between Th17 and Treg cells.

This study aims to investigate the effects of FMT on the JAK/STAT pathway and the Th17/Treg balance, with the goal of providing new insights and therapeutic strategies for ARDS treatment. The study aims to elucidate the mechanisms by which gut microbiota modulates immune regulation in ARDS, enhance the understanding of the disease's pathophysiology, and explore the feasibility of combining FMT with JAK/STAT pathway interventions, potentially offering novel therapeutic strategies for clinical management of ARDS.

## 2 Experimental Section

### 2.1 Construction of Rat ARDS Model and FMT Procedure

Specific Pathogen‐Free (SPF) Sprague‐Dawley (SD) rats (4‐6 weeks old, 200–250g, female rats) were sourced from the Experimental Animal Center of Southwest Medical University. The animals were housed under a 12‐h light/dark cycle, with free access to water and food, and maintained at a temperature of 23–25 °C. Each experimental group includes 6 rats. After one week of acclimatization, the rats were administered a broad‐spectrum antibiotic mixture (100 mg kg^−1^, Sigma, N6386), ampicillin (100 mg kg^−1^, Sigma, A0166), vancomycin (50 mg kg^−1^, Sigma, 861987), and metronidazole (100 mg kg^−1^, Sigma, M3761) via oral gavage for 7 consecutive days.^[^ ^7^ ^]^ Following the antibiotic treatment, the ARDS model was induced by intranasal instillation of lipopolysaccharide (LPS) solution.^[^ ^13^, ^14^ ^]^ During the LPS modeling, the rats were anesthetized with intraperitoneal injection of 7% chloral hydrate (5 mL kg^−1^, Sigma, C2432), and LPS solution (10 mg kg^−1^, Sigma, L2880) was pipetted into a 1 mL pipette (Research Plus series, Eppendorf, Germany). The solution was slowly administered into one nostril to a depth of 0.5–1 cm, while the opposite nostril was temporarily occluded for 1 min to ensure that a high proportion of LPS reached the lungs. Rats in the control group received an equal volume of saline intranasally.

Fresh feces from healthy donor rats were diluted in PBS (Gibco, 14190‐144) at a 1:5 (g mL^−1^) ratio. To ensure the preservation of anaerobic bacteria, L‐cysteine (Sigma–Aldrich, C7352) was added to the suspension as a reducing agent. The fecal suspension was vigorously mixed to achieve homogeneity, followed by filtering through a 0.8–1 mm mesh to remove particulate matter. To further purify the preparation, centrifugation was performed at 800 xg for 3 min to remove undissolved solids, as low‐speed centrifugation helps clear away particulate matter without losing a significant number of bacteria. The suspension was stored at 4 °C and used within 6 h to maintain bacterial viability. The anti‐CD25 + LPS group and anti‐CD25 + LPS + FMT group received intraperitoneal injections of anti‐CD25 antibody (BioLegend, 202114, 300 µg kg^−1^) 10 and 3 days prior to modeling; the JAK + LPS group and JAK + LPS + FMT group received intraperitoneal injections of JAK inhibitor AG490 (Beyotime, S1509, 25 mg kg^−1^) on days 7, 5, and 2 prior to modeling; the control group received an equal volume of saline intranasally. The pre‐application was designed to evaluate their preventive potential against ARDS development.^[^ ^15^ ^]^ 24 h post‐modeling, rats in the FMT groups were administered bacterial suspension (10 ml kg^−1^) via gavage, delivering the fecal suspension directly into the stomach. This ensures that the microbiota is introduced directly into the digestive system, allowing for colonization in the intestines.^[^ ^16^ ^]^ The procedure was performed twice daily for a continuous period of 7 days, with other groups receiving an equal volume of saline via gavage. 24 h after the last FMT, all rats were anesthetized with 7% chloral hydrate (intraperitoneal injection, 5 mL kg^−1^) and then euthanized by cervical dislocation, and tissues were collected for subsequent analyses. Six rats were included in each group. All animal treatment procedures were conducted in accordance with the guidelines proposed by the Animal Ethics Committee of Southwest Medical University (Approval No.: swmu20230067). This study was conducted in strict accordance with the ARRIVE guidelines (https://arriveguidelines.org).

### 2.2 Histopathological Examination of Lung And Intestinal Tissues by He Staining

After cervical dislocation, lung and intestinal tissues were rapidly excised, cut into appropriate sizes, washed with PBS, and fixed in 4% paraformaldehyde (Solarbio, P1110) in a volume 10 times that of the tissue. The tissues were then embedded in paraffin and sectioned at 4 µm thickness. Sections were stained with hematoxylin and eosin (HE) (Solarbio, G1140), deparaffinized, dehydrated, and mounted. Histopathological images were captured under an optical microscope. Nuclei appeared blue, while cytoplasm appeared red or pink.

### 2.3 Flow Cytometry Analysis of Th17/Treg Cell Ratios in Lung and Intestinal Tissues

#### 2.3.1 Cell Collection from Lung and Intestinal Tissues

Rats were euthanized by cervical dislocation, and the small intestine and lung tissues were exposed and collected, placed in pre‐chilled PBS. The small intestine was rinsed multiple times with pre‐chilled PBS, cut into small pieces, and digested with 5 mm EDTA (Thermo Fisher Scientific, 15575020) at 37 °C for 20 min with shaking, then the supernatant was collected and mixed with stop solution. The remaining intestinal tissue was washed with PBS, cut into 1 mm^3^ fragments, and digested with Type I collagenase (Sigma–Aldrich, C0130) at 37 °C for 30 min with shaking. The digested cell suspension was pipetted and the supernatant collected, which was then mixed with the stop solution. Both supernatants were filtered through a 100 µm cell strainer and centrifuged at 400 xg for 10 min to collect the cells. Lung tissue was processed similarly, cut into 1 mm^3^ fragments, and digested with Type I collagenase at 37 °C for 50 min with shaking. The digested cell suspension was pipetted to collect the supernatant, which was then mixed with stop solution, filtered, and centrifuged at 400 xg for 10 min to collect the cells. Lymphocytes from lung and intestinal tissues were isolated according to the manufacturer's instructions for the TBD Company's Rat Organ Lymphocyte Separation Medium (LTS1083P).

#### 2.3.2 Detection of Treg and Th17 Cells

To detect Treg cells (CD25^+^FOXP3^+^), the isolated lymphocytes from small intestine and lung tissues were adjusted to a density of 2 × 10^7^/mL. 100 µL of cell suspension was incubated with 0.5 µg of CD25 antibody (BioLegend, 202114) at room temperature for 20 min in the dark. The cells were then washed with Flow Cytometry Staining Buffer (BioLegend, 420201) and incubated with Fix & Perm Kit working solution (LinkoCare, GAS003/2) at room temperature for 60 min. After incubation, without further washing, the cells were washed with permeabilization buffer, resuspended, and incubated with 5 µL of Foxp3 antibody (eBioscience, 12‐5773‐82) at room temperature for 30 min in the dark. The cells were washed with permeabilization buffer and resuspended in Flow Cytometry Staining Buffer for flow cytometry analysis (NovoCyte, ACEA). To detect Th17 cells (CD4⁺IL‐17A⁺), 1 mL of cell suspension was incubated with PMA/Ionomycin mixture (1X) (LinkoCare, CS1001) and BFA/Monensin Mixture (1X) (LinkoCare, CS1002) at 37 °C in a 5% CO_2_ incubator for 12 h. 100 µL of cell suspension was incubated with 0.25 µg of CD4 antibody (BioLegend, 201505) at room temperature for 20 min, followed by the addition of 100 µL of MEDIUM A and incubation at room temperature for 15 min. The cells were washed with Flow Cytometry Staining Buffer and then incubated with 100 µL of MEDIUM B and 0.125 µg of IL‐17A antibody (BioLegend, 17‐7177‐81) at room temperature for 20 min. The cells were washed with Flow Cytometry Staining Buffer and resuspended for flow cytometry analysis.

### 2.4 Quantitative PCR (qPCR)

Total RNA was extracted from lung and intestinal tissues for reverse transcription and qPCR analysis to assess the expression levels of Foxp3 and RORγt mRNA. Total RNA was extracted using RNAiso Plus (Takara, 9109) according to the manufacturer's instructions, mixed with chloroform, centrifuged at 12,000 xg for 15 min, and the supernatant was collected. RNA was precipitated with isopropanol (Thermo Fisher Scientific, A461‐1), washed with 75% ethanol, and then dissolved in RNA‐free water. The concentration was measured, and the RNA was stored. Reverse transcription was performed using the PrimeScript^TM^ RT reagent Kit with gDNA Eraser (Perfect Real Time, Takara, RR047A), following the manufacturer's instructions for reaction setup and execution. cDNA synthesis was performed using the reverse transcription product. qPCR was performed using PerfectStart Green qPCR SuperMix (AQ601‐02, TransGen Biotech). The reaction mixture consisted of 2 × SuperMix, specific primers (10 µm), and cDNA template, with a final volume of 10 µL. The PCR program was set at 94 °C for 30 s, followed by 40 cycles of 94 °C for 5 s and 60 °C for 30 s. Primer sequences are listed in **Table** **1**. Data were analyzed using the 2^−ΔΔCt^ relative quantification method.

**Table 1:** qPCR primer sequences.

Primer Name	Sequence (5′–3′)
Foxp3‐F	CACCTGGCTGGGAAGATGG
Foxp3‐R	CTCCGCACAGCAAACAAGC
RORC‐F	TGAAAGCAGGAGCAATGGAA
RORC‐R	CCTCAGAAAAACACAGGGCG
GAPDH‐F	GGCACAGTCAAGGCTGAGAATG
GAPDH‐R	ATGGTGGTGAAGACGCCAGTA

#### 2.4.1 Western Blot

Total protein was extracted from lung tissues using RIPA lysis buffer (Beyotime, P0013B) supplemented with protease inhibitors (Roche, 04693132001) and incubated on ice for 30 min. The lysate was centrifuged at 16,100 xg for 15 min, and the supernatant was collected. Protein concentration was determined using the BCA Protein Assay Kit (Thermo, 23225) according to the manufacturer's protocol. Thirty micrograms of protein per sample were separated by 10% SDS‐PAGE and transferred to a PVDF membrane (Millipore, IPVH00010). The membrane was blocked with 5% non‐fat milk at room temperature for 1 h and then incubated with the following primary antibodies overnight: JAK1 (proteintech, 66466‐1‐Ig, 1:3000), JAK2 (HUBIO, M1501‐8, 1:2000), p‐JAK1 (abcam, ab138005, 1:1000), p‐JAK2 (abcam, ab195055, 1:1000), STAT3 (HUBIO, ET1607‐38, 1:2000), STAT5 (proteintech, 13179‐1‐AP, 1:1000), p‐STAT3 (HUBIO, ET1603‐40, 1:5000), p‐STAT5 (ABClonal, AP0887, 1:500), RORγt (HUBIO, ab140807, 1:1000), Foxp3 (proteintech, ab309108, 1:1000) and GAPDH (abcam, ab181602, 1:10000). Afterward, the membrane was washed three times with TBST for 10 min each, followed by incubation with HRP‐conjugated secondary antibody (Proteintech, SA00001‐2, 1:5000) at room temperature for 1 h. The membrane was then washed three more times with TBST for 10 min each. The membrane was then developed using ECL chemiluminescence reagent (Thermo, 32109) and imaged on a Bio‐Rad ChemiDoc MP imaging system. Protein band quantification was performed using ImageJ software. All experiments were repeated three times.

### 2.5 Enzyme‐Linked Immunosorbent Assay (ELISA) for Serum Inflammatory Cytokines

Serum samples from each group of rats were collected and used to measure the levels of IL‐2 (Elabscience, E‐EL‐R0013), IL‐6 (Elabscience, E‐EL‐R0015), IL‐8 (Elabscience, E‐EL‐R0566), IL‐10 (Elabscience, E‐EL‐R0016), IL‐17A (Elabscience, E‐EL‐R0566), IL‐23 (Elabscience, E‐EL‐R0569), IL‐35 (BioLegend, 434904), and TGF‐β1 (Elabscience, E‐EL‐0162) using ELISA kits, according to the manufacturer's instructions.

### 2.6 Statistical Analysis

Data were analyzed using Microsoft Excel and GraphPad Prism 9.0.0. Continuous variables are presented as mean ± standard error of the mean (SEM). Statistical significance was evaluated using two‐way ANOVA for experiments involving more than one independent and/or dependent variable. One‐way ANOVA was used for experiments with a single independent variable affecting a continuous outcome. Sidak's post‐hoc test was employed for multiple comparisons in two‐way ANOVA setups, while Tukey's post‐hoc test was used following one‐way ANOVA. All statistical tests were two‐tailed, and a *P*‐value less than 0.05 was considered statistically significant. The correlation was analyzed using the Spearman rank correlation coefficient.

### 2.7 Ethics Statement

All procedures in this study were conducted in accordance with the guidelines of the National Institutes of Health and the ARRIVE guidelines (https://arriveguidelines.org) and were approved by the Animal Ethics Committee of Southwest Medical University (Approval No: swmu20230067).

## 3 Results

### 3.1 FMT Alleviates LPS‐Induced ARDS Damage in Rat Lung and Intestinal Tissues

**Figure 1:**
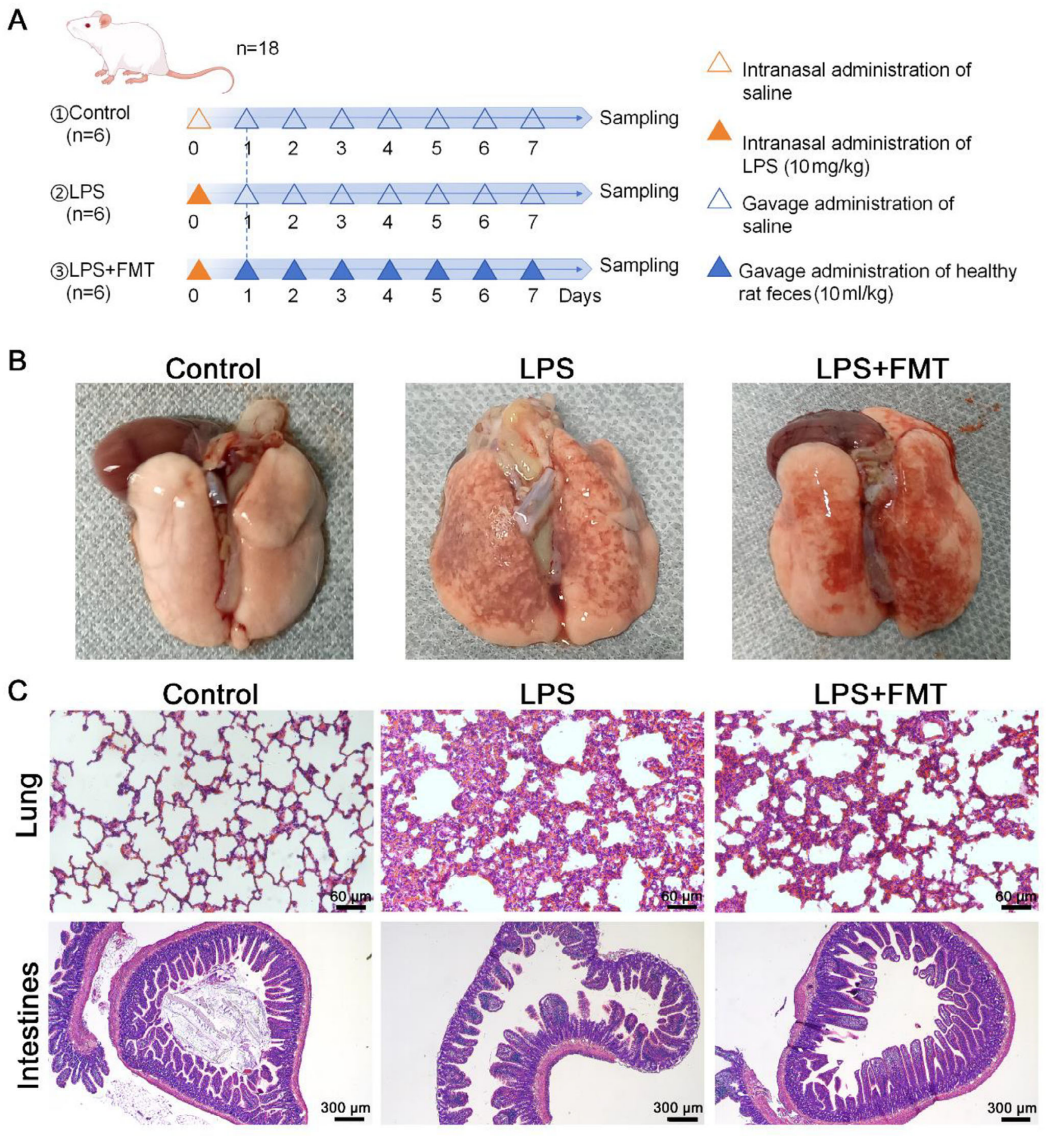
FMT alleviates damage in the lung and intestinal tissues of ARDS rats. A) Schematic diagram of model construction. B) Photographs of lung tissues. C) HE staining of lung tissues (200×), intestinal tissue (400×). Each experimental group includes 6 rats.

Rats in the control, LPS, and LPS + FMT groups were treated with saline, LPS, and fecal microbiota suspension from healthy rats, respectively, via gavage at different time points. Samples were collected on day 8 (**Figure** **1**A). Photographs of lung tissues showed a normal appearance in the control group, significant congestion and hemorrhage in the LPS group, and improved lung damage in the LPS + FMT group (Figure 1B). Histopathological examination of lung and intestinal tissues using HE staining revealed normal structures in the control group. In the LPS model group, lung tissues showed alveolar structure destruction, thickened alveolar walls, and significant inflammatory cell infiltration. Intestinal tissues displayed shortened and widened villi, disordered epithelial cell arrangement, and mild inflammatory cell infiltration. In the FMT+LPS group, both lung and intestinal tissues showed significant pathological improvement, with clearer alveolar spaces, reduced inflammatory infiltration, more normal villi morphology, and restored epithelial cell alignment (Figure 1C). These results indicate that FMT has a mitigative effect on lung and intestinal tissue damage in ARDS rats.

### 3.2 FMT Restores Th17/Treg Cell Balance in LPS‐Induced ARDS Rat Model

**Figure 2:**
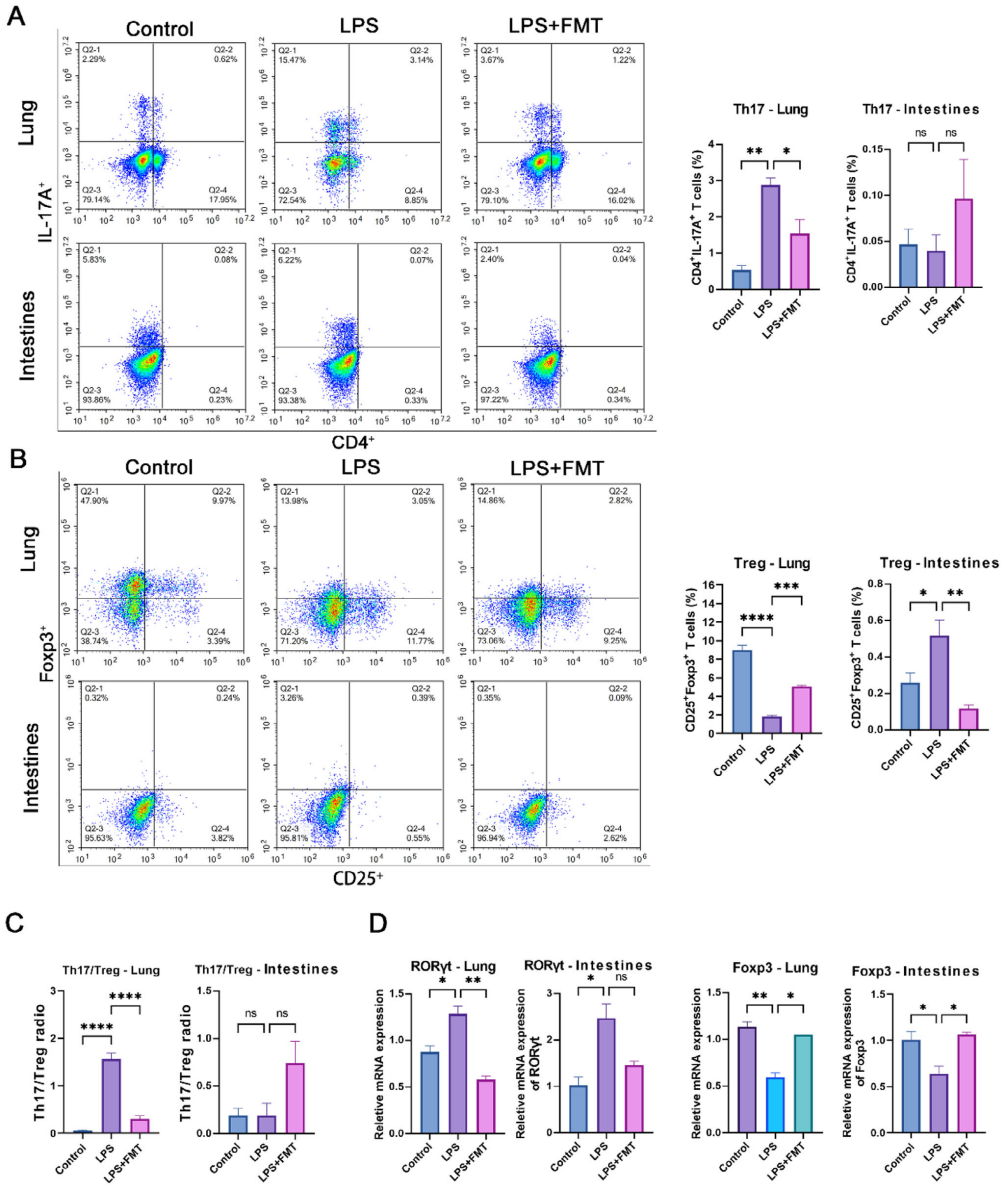
FMT restores Th17/Treg cell balance in LPS‐induced ARDS rat model. A) Changes in the proportion of Th17 cells (CD4^+^ IL‐17A^+^) in lung and intestinal tissues. B) Changes in the proportion of Treg cells (CD25^+^ Foxp3^+^) in lung and intestinal tissues. C) Th17/Treg cell ratio in lung and intestinal tissues. D) Changes in Foxp3 and RORγt mRNA in lung and intestinal tissues. Data are presented as mean ± SEM, with all graphs representing results from at three independent experiments. Each experimental group included 6 rats. Significant differences between groups were assessed using one‐way ANOVA followed by Tukey's post‐hoc test. ^*^ *p* < 0.05, ^**^ *p* < 0.01, ^***^ *p* < 0.001, ^****^ *p* < 0.0001, “ns” represents no significant difference.

Flow cytometry was used to analyze the proportion of Th17 cells (CD4^+^IL‐17A^+^) and Treg cells (CD25^+^Foxp3^+^) in the lung and intestinal tissues of rats. The results showed that in the lung tissues, the proportion of Th17 cells significantly increased in the LPS group and significantly decreased after FMT treatment. No significant differences were observed in the intestinal tissues (**Figure** **2**A). For Treg cells, a significant reduction was observed in the LPS group in the lung tissues, which significantly increased after FMT treatment (Figure 2B). Additionally, compared to the control group, the Th17/Treg ratio significantly increased in the lung tissues of the LPS group and significantly decreased after FMT treatment. In the intestinal tissues, the Th17/Treg ratio showed no significant change in both the LPS group and after FMT treatment compared to the control group (Figure 2C). In terms of gene expression, RORγt mRNA significantly increased in the lungs of the LPS group and significantly decreased with FMT treatment; Foxp3 mRNA expression significantly decreased in the LPS group and significantly increased following FMT treatment (Figure 2D). These findings demonstrate that FMT effectively restores the balance of Th17/Treg cells in the lung tissues of rats with LPS‐induced ARDS, influencing this balance by modulating the expression of Foxp3 and RORγt mRNA.

### 3.3 Effects of Different Treatments on the Expression of Proteins and Transcription Factors Related to the JAK/STAT Signaling Pathway

**Figure 3:**
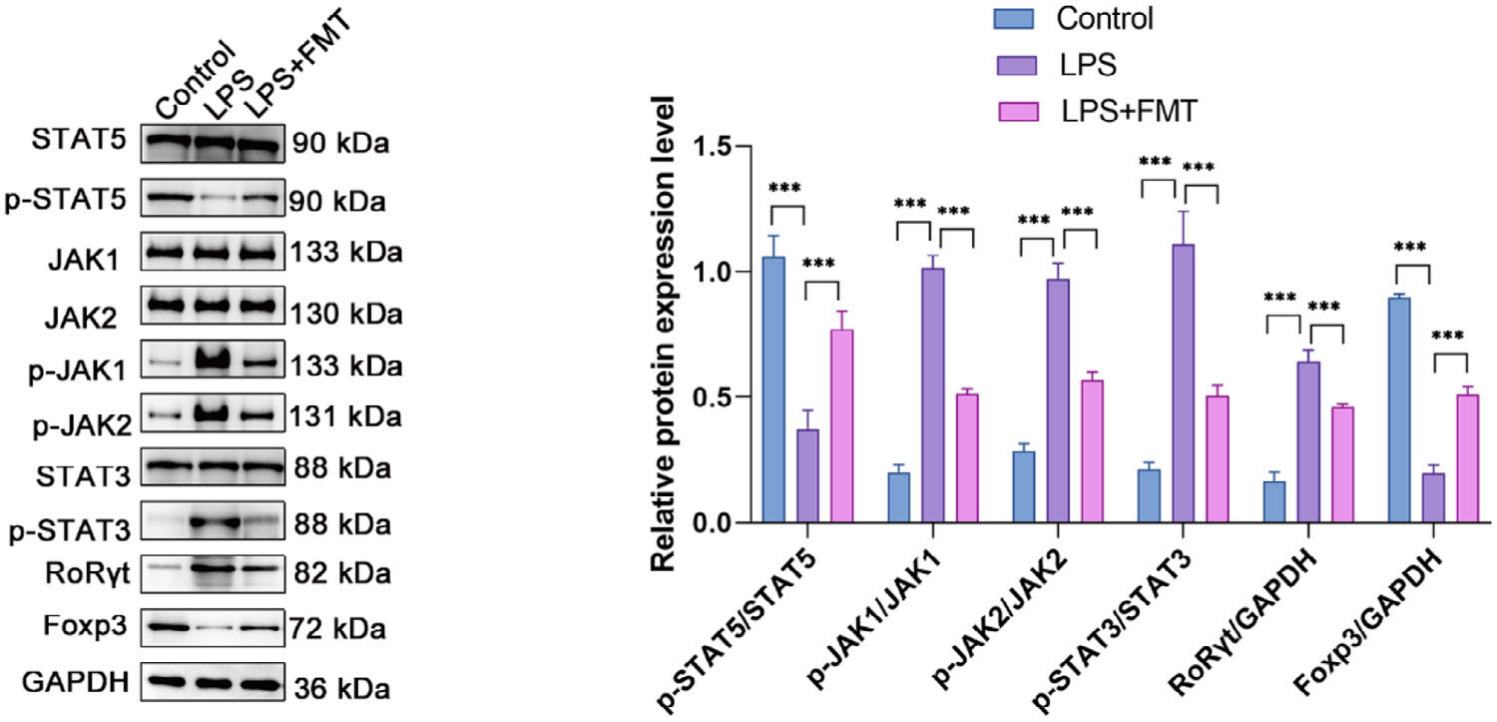
Effects of different treatments on the expression of proteins and transcription factors related to the JAK/STAT signaling pathway. Western blot analysis was performed to detect the expression of various proteins and their phosphorylated forms, including STAT5 and its phosphorylated form p‐STAT5, JAK1 and its phosphorylated form p‐JAK1, JAK2 and its phosphorylated form p‐JAK2, STAT3 and its phosphorylated form p‐STAT3, transcription factors RoRγt and Foxp3, as well as the internal control GAPDH. Data are shown as mean ± SEM, with all graphs representing results from three independent experiments. Each experimental group includes 6 rats. Statistical differences were determined using one‐way ANOVA with Tukey's post‐hoc test. ^*^ *p <* 0.05, ^**^ *p <* 0.01, ^***^ *p* < 0.001, “ns” represents no significant difference.

Western blot analysis showed that, compared with the control group, the relative expression level of p‐STAT5/STAT5 was significantly decreased in the LPS‐treated group, while the relative expression levels of p‐JAK1/JAK1, p‐JAK2/JAK2, and p‐STAT3/STAT3 were significantly increased. Furthermore, LPS + FMT treatment significantly increased the relative expression level of p‐STAT5/STAT5 compared to LPS treatment and significantly decreased the relative expression levels of p‐JAK1/JAK1, p‐JAK2/JAK2, and p‐STAT3/STAT3. LPS treatment significantly increased the relative expression level of RoRγt/GAPDH and decreased the relative expression level of Foxp3/GAPDH. In contrast, in the LPS + FMT group, the relative expression level of RoRγt/GAPDH was significantly reduced, and the relative expression level of Foxp3/GAPDH was significantly increased (**Figure** **3**).

### 3.4 FMT and JAK Inhibitors Improve Lung Tissue Morphology to Mitigate LPS‐Induced ARDS

**Figure 4:**
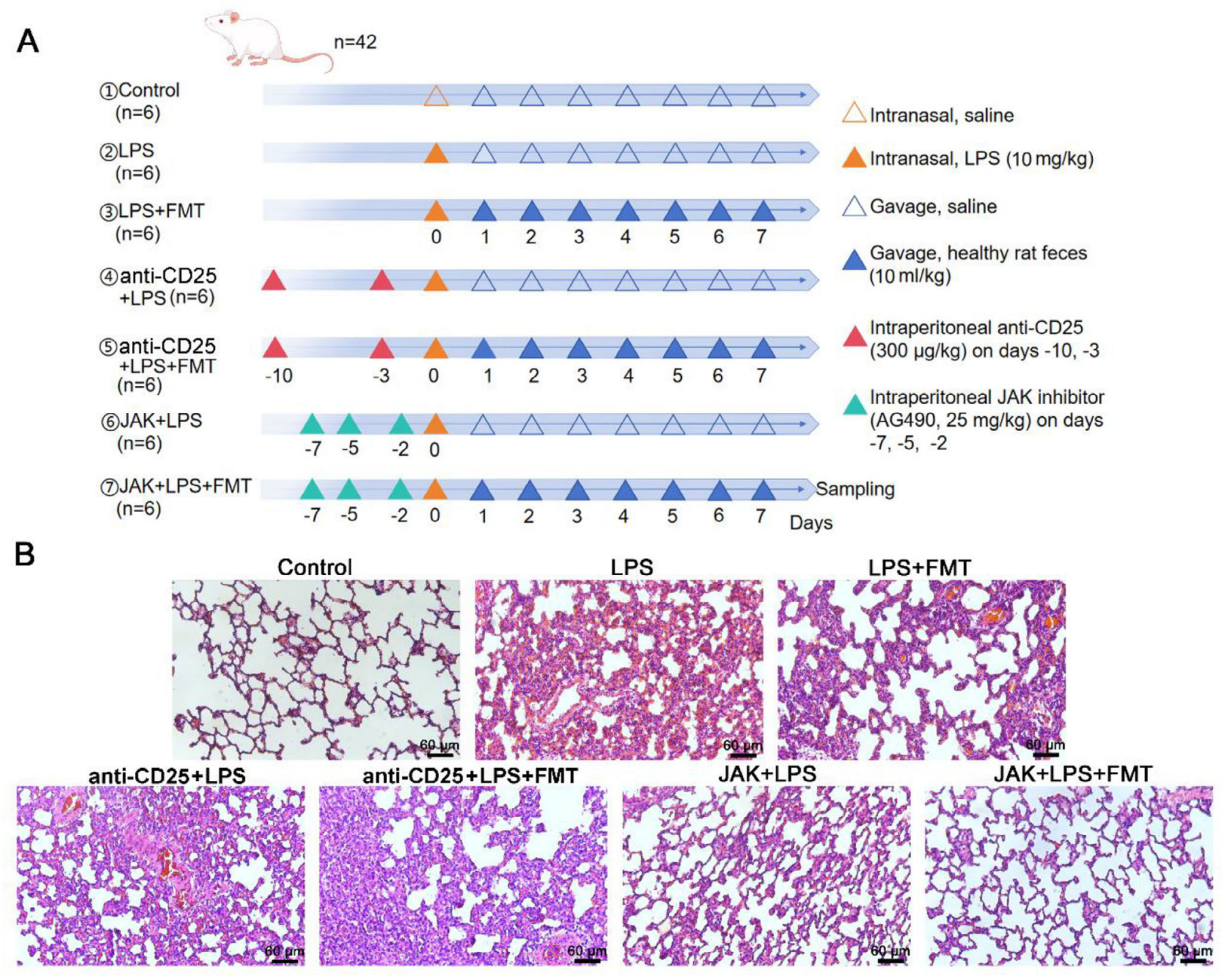
FMT and JAK inhibitors improved lung tissue morphology and alleviate LPS‐induced ARDS. A) Experimental design schematic. B) HE staining of lung tissues. An ARDS model was established by intranasal administration of LPS, followed by gavage of fecal suspension from healthy rats for FMT. Intraperitoneal injection of anti‐CD25 antibody and JAK inhibitors was administered to the different groups of rats, with samples collected 24 h after the last FMT. Each experimental group includes 6 rats.

The experiment included seven groups, with six rats in each group, treated differently (**Figure** **4**A). HE staining of lung tissues revealed normal tissue structure in the control group, significant inflammation and damage in the LPS group, and a reduction in damage in the LPS + FMT group compared to the LPS group. Treatment with the depletion agent anti‐CD25 antibody resulted in slightly increased tissue damage compared to the LPS group. However, damage was alleviated after FMT treatment in the anti‐CD25 + LPS group. JAK inhibitor treatment led to lighter tissue damage in the JAK + LPS group compared to the LPS group. Further structural restoration and near‐normal lung tissue morphology were observed after FMT treatment (Figure 4B).

### 3.5 FMT Restores Serum Inflammatory Cytokine Levels in ARDS Rats Induced by Treg Cell Depletion and JAK Inhibition

**Figure 5:**
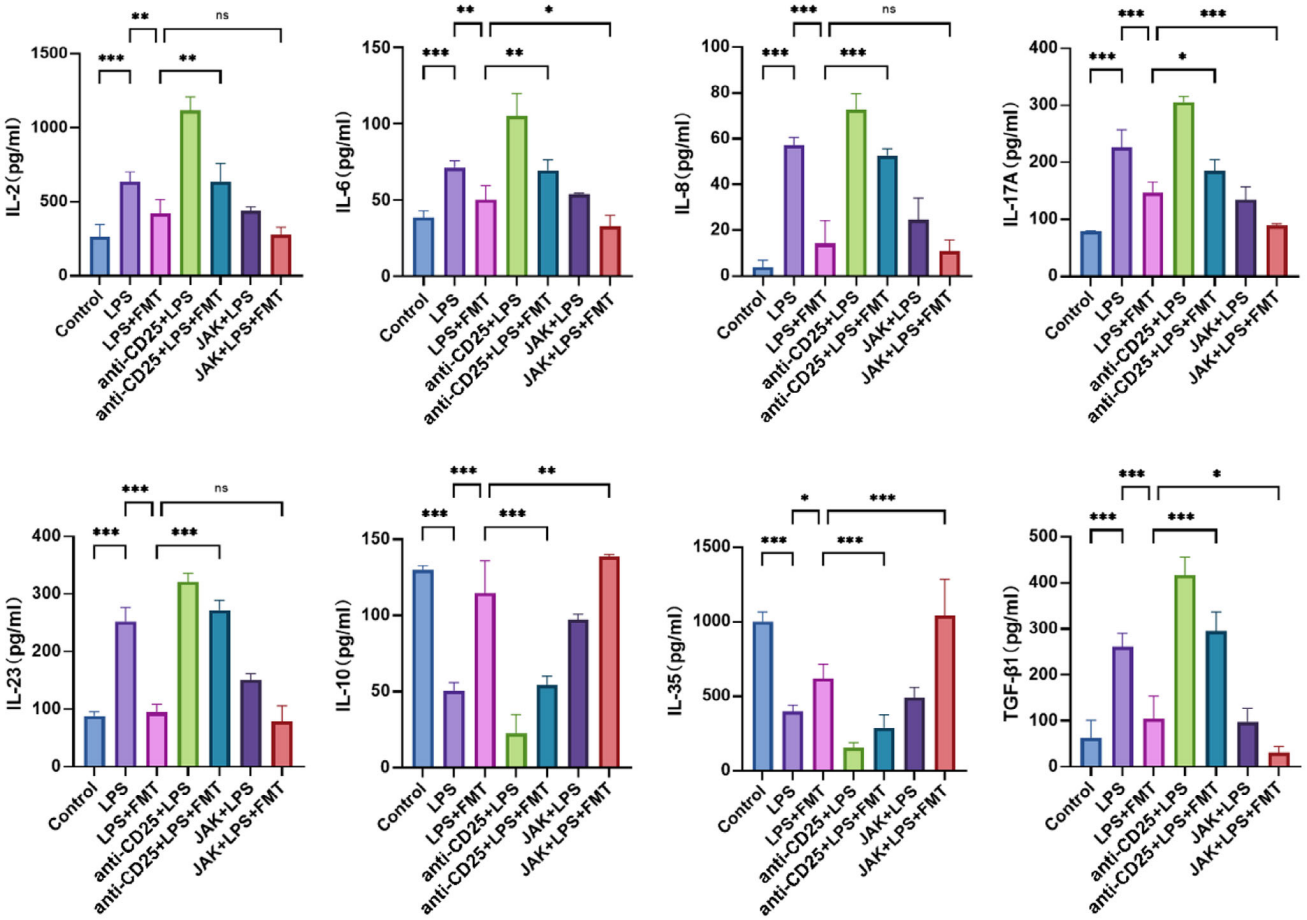
FMT reverses serum inflammatory cytokine changes caused by Treg depletion and JAK inhibitors in ARDS rats. Serum levels of IL‐2, IL‐6, IL‐8, IL‐17A, IL‐23, IL‐10, IL‐35, and TGF‐β1 were measured and compared among the experimental groups using ELISA. Data are shown as mean ± SEM, with all graphs representing results from three independent experiments. Each experimental group includes 6 rats. Statistical differences were determined using one‐way ANOVA with Tukey's post‐hoc test. ^*^ *p* < 0.05, ^**^ *p* < 0.01, ^***^ *p* < 0.001, “ns” represents no significant difference.

ELISA results showed significant differences in serum inflammatory cytokine levels among the rat groups. Compared to the control group, the LPS group showed significantly elevated levels of inflammatory cytokines, including IL‐2, IL‐6, IL‐8, IL‐17A, IL‐23, and TGF‐β1. These cytokine levels significantly decreased after FMT treatment. The addition of the depletion agent anti‐CD25 antibody reversed the FMT‐induced reductions in inflammatory cytokine levels. Treatment with JAK inhibitors further reduced these cytokine levels, with significant decreases in IL‐6, IL‐17A, and TGF‐β1. Conversely, the levels of anti‐inflammatory cytokines IL‐10 and IL‐35 showed an opposite trend. Compared to the control group, levels of IL‐10 and IL‐35 were significantly reduced in the LPS group, but significantly increased after FMT. The addition of anti‐CD25 antibody also reversed the increases in IL‐10 and IL‐35 levels caused by FMT. Subsequent treatment with JAK inhibitors further enhanced the levels of IL‐10 and IL‐35 (**Figure** **5**).

### 3.6 FMT Modulates Th17/Treg Cell Balance to Mitigate Pulmonary Inflammation

**Figure 6:**
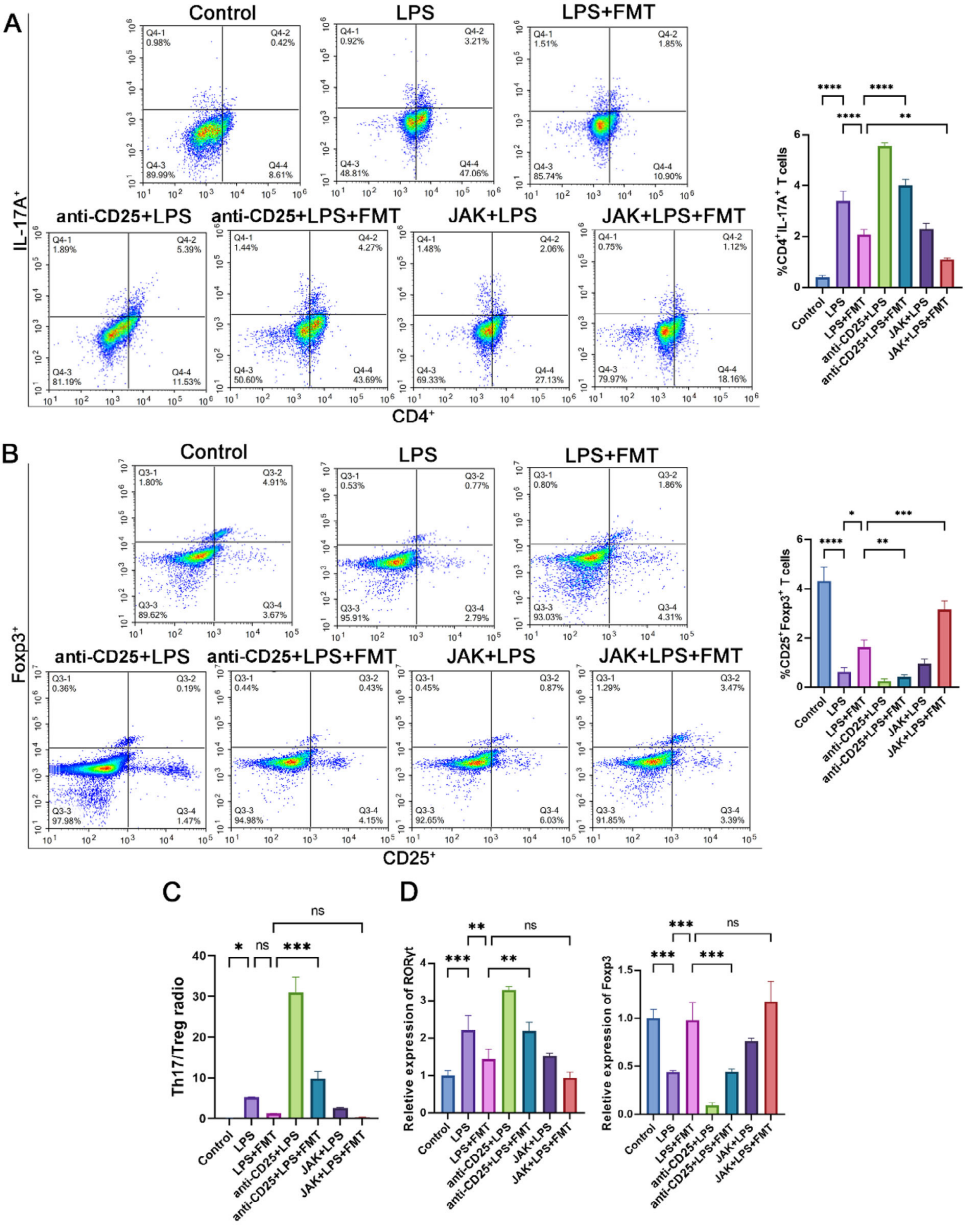
FMT modulates Th17/Treg cell balance in lung tissue. A) Changes in the proportion of Th17 cells (CD4^+^IL‐17A^+^) detected by flow cytometry. B) Changes in the proportion of Treg cells (CD25^+^Foxp3^+^) detected by flow cytometry. C) Th17/Treg cell ratio in lung tissue. D) Expression levels of Foxp3 and RORγt mRNA measured by qPCR. Data are shown as mean ± SEM, with all graphs representing results from three independent experiments. Each experimental group includes 6 rats. Statistical differences were determined using one‐way ANOVA with Tukey's post‐hoc test. ^*^ *p <* 0.05, ^**^ *p <* 0.01, ^***^ *p <* 0.001, ^****^ *p <* 0.0001, “ns” represents no significant difference.

Flow cytometry results demonstrated that, compared to the control group, the proportion of Th17 cells significantly increased in the LPS group, significantly decreased after FMT treatment, increased with the depletion agent anti‐CD25 antibody, and was inhibited by JAK inhibitor treatment (**Figure** **6**A). Conversely, the proportion of Treg cells was significantly reduced in the LPS group compared to the control, significantly increased after FMT treatment, further reduced with anti‐CD25 antibody treatment, and increased with JAK inhibitor treatment (Figure 6B). Meanwhile, the Th17/Treg ratio significantly increased in the LPS group, decreased after FMT treatment, increased further upon Treg cell depletion, and decreased after JAK inhibitor treatment (Figure 6C).

**Figure 7:**
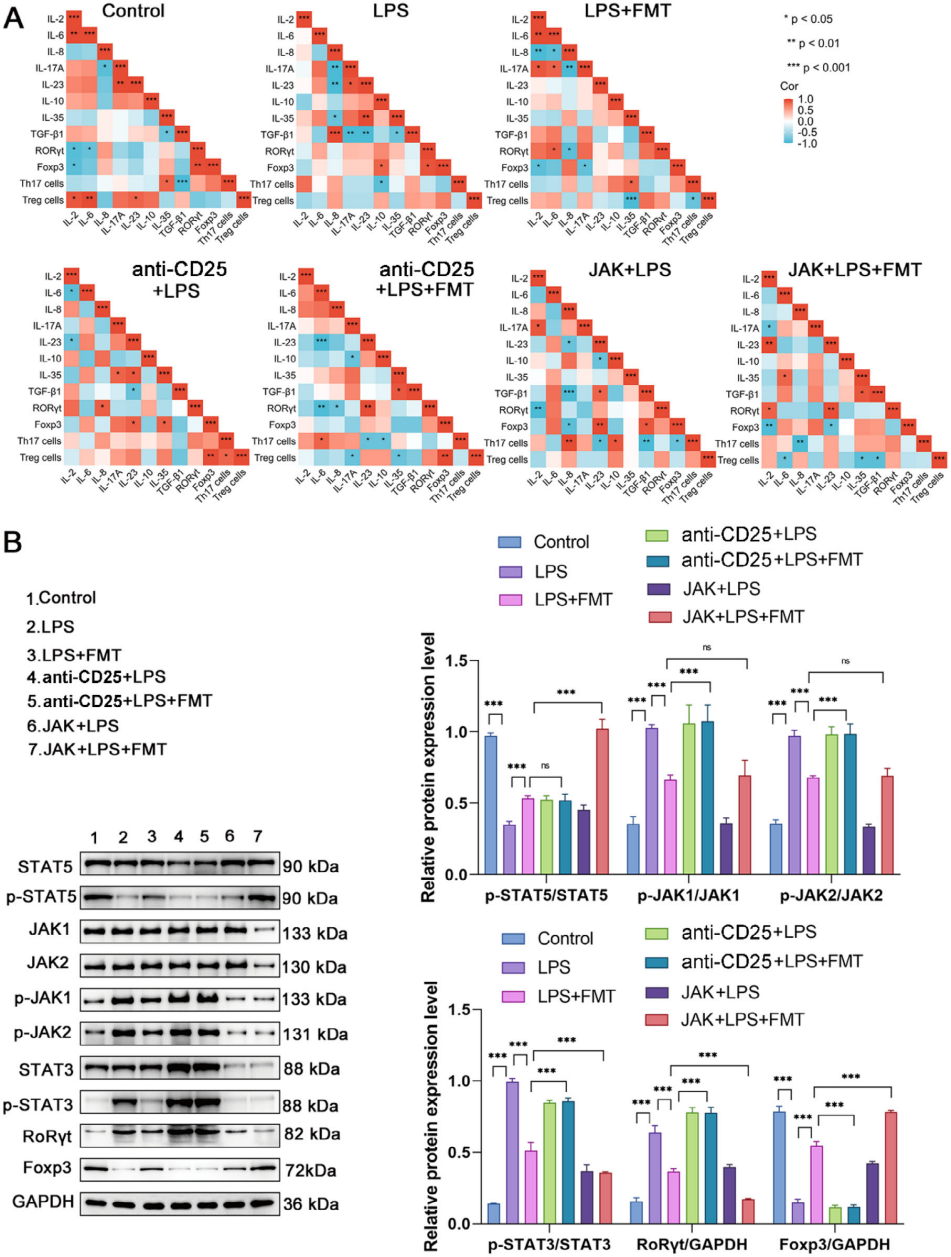
Regulatory Impact of Immunomodulatory T Cells and JAK Pathway Inhibitors on LPS‐Induced Inflammatory Responses. A) Correlations were analyzed among IL‐2, IL‐6, IL‐8, IL‐17A, IL‐23, IL‐10, IL‐35, TGF‐β1, RORγt, Foxp3, Th17 cells, and Treg cells in Control, LPS, LPS + FMT, anti‐CD25 + LPS, anti‐CD25 + LPS + FMT, JAK + LPS, and JAK + LPS + FMT groups. Coefficients are color‐coded with red indicating positive and blue indicating negative correlations. B) Evaluating the effects of LPS, Treg cell depletion agents, JAK inhibitors, and FMT (alone or in combination) on the phosphorylation and expression of STAT5, JAK1, JAK2, STAT3, RoRγt, and Foxp3 via Western blot. Data are shown as mean ± SEM, with all graphs representing results from three independent experiments. Each experimental group includes 6 rats. Statistical differences were determined using one‐way ANOVA with Tukey's post‐hoc test. ^*^ *p* < 0.05, ^**^ *p* < 0.01, ^***^ *p* < 0.001, “ns” represents no significant difference.

qPCR results showed that RORγt mRNA significantly increased and Foxp3 mRNA significantly decreased in the LPS group, with opposite changes after FMT treatment. Treg cell depletion reversed the mRNA expression changes induced by FMT, while JAK inhibitor treatment showed no significant changes (Figure 6D). Western blot analysis of lung tissues for Foxp3 and RORγt protein levels corroborated the mRNA results, with no significant changes in Foxp3 protein levels before and after Treg cell depletion and JAK inhibitor treatment (**Figure** **7**B).

### 3.7 Regulatory Impact of Immunomodulatory T Cells and JAK Pathway Inhibitors on LPS‐Induced Inflammatory Responses

Spearman correlation analysis was performed to examine the relationships serum inflammatory cytokines (IL‐2, IL‐6, IL‐8, IL‐17A, IL‐23, IL‐10, IL‐35, TGF‐β1) and the expression of RORγt, Foxp3 proteins, as well as the proportions of Th17 and Treg cells. The analysis showed significant correlations in different treatment groups. In the LPS group, IL‐8 was positively correlated with TGF‐β1, IL‐23 with IL‐35, and IL‐10 with Foxp3. In contrast, IL‐8 was negatively correlated with IL‐17A and IL‐23, and IL‐35 with TGF‐β1. In the LPS + FMT group, significant positive correlations were observed between IL‐2 and IL‐6, and IL‐6 and RORγt, Negative correlations were observed between IL‐2 and Foxp3, and IL‐17A and Foxp3. In the anti‐CD25 + LPS + FMT group, IL‐6 was positively correlated with the Th17 cell ratio, while IL‐17A negatively correlated with the Treg cell ratio. In the JAK + LPS + FMT group, IL‐2 was positively correlated with RORγt. IL‐6 was negatively correlated with Foxp3 and the Treg cell ratio (Figure **7**A). These results suggest that FMT and JAK inhibitors can restore immune balance and mitigate inflammatory responses by regulating cytokine interactions. Treg cell depletion and JAK inhibitors can reverse the effects of FMT on inhibiting Th17 cells and promoting Treg cells.

Western blot analysis showed decreased p‐STAT3, p‐JAK1, and p‐JAK2 but increased p‐STAT5 in the LPS + FMT group compared to the LPS group. However, Treg cell depletion and JAK inhibitors reverse this suppression, as evidenced by the restoration of phosphorylation levels in the anti‐CD25 + LPS + FMT and JAK + LPS + FMT groups. Statistical analysis confirmed that both interventions significantly restored p‐STAT5/STAT5, p‐STAT3/STAT3, p‐JAK1/JAK1, and p‐JAK2/JAK2 levels. These results demonstrate their ability to counteract the inhibitory effects of FMT on the JAK/STAT pathway (Figure 7B).

## 4 Discussion

ARDS is a severe, progressive form of respiratory failure caused by various etiologies, characterized by significant alveolar‐capillary barrier damage leading to pulmonary edema and acute inflammatory responses, which severely impair lung function.^[^ ^17^ ^]^ Despite the complex and multifactorial pathophysiology of ARDS, inflammation plays a pivotal role in its onset and progression. Recent studies suggest that the composition of the pulmonary microbiota in critically ill patients can predict clinical outcomes, and interactions between the gut‐lung axis may play a role in the pathogenesis of ARDS by influencing systemic inflammation and immune regulation.^[^ ^18^, ^19^ ^]^ In ARDS, gut dysbiosis is marked by an increase in harmful bacteria such as Enterobacteriaceae and Proteobacteria, which are associated with a reduction in microbial diversity.^[^ ^20^ ^]^ This microbial imbalance promotes gut permeability and bacterial translocation, exacerbating systemic inflammation and lung injury, thus contributing to the pathogenesis of ARDS.^[^ ^21^ ^]^ Furthermore, the gastrointestinal microbiota of ARDS patients has been shown to induce neuroinflammation and cognitive dysfunction in mouse models, potentially through the gut‐lung axis affecting central nervous system inflammation. This further supports the link between gut microbiota and the pathophysiology of ARDS.^[^ ^22^ ^]^ However, the specific mechanisms by which FMT alleviates ARDS remain unclear. This study focuses on the relationship between FMT and ARDS pathology, aiming to explore the mitigative effects of FMT on lung and intestinal tissue damage in an LPS‐induced ARDS rat model and to reveal its mechanisms in regulating immune balance and inflammatory signaling pathways. The study found that FMT treatment significantly improved lung and intestinal tissue damage in the LPS‐induced ARDS model, evidenced by reduced alveolar structure destruction, decreased inflammatory cell infiltration, and shortened intestinal villi, with notable restoration of lung and intestinal tissue architecture. These findings may involve FMT's protective effects on pulmonary epithelial and endothelial cell functions, which play crucial roles in maintaining the alveolar barrier and reducing extracellular matrix destruction.

Recent studies have shown that an imbalance in the Th17/Treg cell ratio plays a significant role in the pathogenesis of ARDS.^[^ ^10^ ^]^ Th17 cells, a subset of CD4^+^ T cells regulated by RORγt, are crucial in autoimmune diseases through their secretion of IL‐17A and IL‐17F.^[^ ^23^ ^]^ Conversely, the expression level of Foxp3 is closely linked to Treg cells functionality; its downregulation decreases the proportion of Treg cells and impairs their immunoregulatory capacity.^[^ ^24^, ^25^ ^]^ This imbalance in Th17/Treg cells results in the overproduction of pro‐inflammatory cytokines such as IL‐6 and IL‐17, exacerbating lung inflammation and tissue damage.^[^ ^26^ ^]^ This study found that FMT significantly restored the Th17/Treg cell balance in the lung tissues of rats with LPS‐induced ARDS, though it had less significant effects in intestinal tissues. Further analysis showed that upregulation of RORγt is associated with an increased proportion of Th17 cells and intensified inflammatory responses, as observed by Lückel et al.^[^ ^27^ ^]^ The downregulation of Foxp3, meanwhile, correlates with a reduction in the proportion of Treg cells and a weakening of immune regulation. Additionally, by modulating the expression of Foxp3 and RORγt, FMT reversed the LPS‐induced increase in the Th17 cell ratio and the decrease in the Treg cell ratio, thereby restoring the Th17/Treg cell balance in the LPS‐induced ARDS rat model. This highlights the potential role of gut microbiota regulation in the treatment of ARDS. FMT has been shown to restore Treg homeostasis and alleviate inflammation by modulating the PD‐1/PD‐L1 signaling pathway, enhancing Treg accumulation, and regulating gut microbiota, as observed in a neonatal mouse model of allergic asthma.^[^ ^28^ ^]^

Activation of the JAK/STAT3 signaling pathway is closely associated with the pathological progression of ARDS.^[^ ^29^ ^]^ This pathway exacerbates ARDS inflammation by regulating the production of inflammatory cytokines and promoting inflammatory cell differentiation and function.^[^ ^30^ ^]^ When activated, phosphorylated STAT3 enters the nucleus, enhancing the expression of RORC and IL‐17A, thereby promoting the development and function of Th17 cells.^[^ ^31^ ^]^ This study reveals for the first time that FMT exerts its protective effects by inhibiting the JAK/STAT3 signaling pathway and modulating the Th17/Treg cell ratio. Specifically, FMT inhibited the activation of the JAK/STAT pathway in lung tissues, reducing the inflammation induced by LPS. Experimental data from Treg cell depletion and JAK inhibitor interventions show that Treg cell depletion reversed the reduction in pro‐inflammatory cytokine levels and Th17 cell ratio caused by FMT. It also resulted in a decrease in anti‐inflammatory cytokine levels and Treg cell ratio. JAK inhibitors further enhanced the changes in inflammatory cytokine levels and the Th17/Treg cell ratio induced by FMT. This indicates the crucial role of Treg cells in regulating inflammation in the ARDS model. JAK inhibitor treatment exhibited significant anti‐inflammatory effects by reducing pro‐inflammatory cytokine levels and inhibiting activation of the JAK/STAT pathway, thereby alleviating tissue damage. Moreover, Treg cell depletion reversed the reductions in RORγt mRNA and protein expression levels induced by FMT, suggesting that RORγt may exacerbate inflammatory responses by regulating the expression of pro‐inflammatory factors and enhancing Th17 cell activity. This aligns with findings by Bernard‐Raichon et al., who demonstrated that inducing RORγt + Tregs could reduce pulmonary inflammation, further supporting the importance of regulating RORγt in controlling lung inflammation.^[^ ^32^ ^]^ FMT has shown promise in treating conditions such as recurrent *Clostridioides difficile* infection, but its use in critically ill patients, including those with sepsis and ARDS, poses significant risks. In sepsis, dysregulated immune responses and gut microbiota disruptions can worsen the condition. Although FMT may help restore microbiota balance and immune function, it has been linked to severe complications such as bacteremia and other infections.^[^ ^33^ ^]^ The risk is compounded by the variability in screening practices for pathogens in FMT preparations, with reports of multidrug‐resistant organisms and other dangerous pathogens being transmitted.^[^ ^34^ ^]^ Therefore, while FMT holds therapeutic potential, its application in these high‐risk populations remains investigational and warrants careful consideration and more rigorous safety protocols.^[^ ^35^ ^]^

The limitations of this study include the fact that, although significant experimental results were achieved in the rat model, the direct applicability of these findings to clinical therapy requires further validation. Additionally, this study only explored the effects of FMT and JAK inhibitors during the acute inflammatory phase without assessing their long‐term effects and potential side effects. Future research should incorporate more diverse models and clinical trials to comprehensively evaluate the safety and efficacy of these treatment methods. Study showed that the presence of mucosa‐associated lymphoid tissue plays a crucial role in immune responses, stimulating the production of IFN‐γ and TH1 cytokines, or promoting anti‐inflammatory cytokine production by Th2 lymphocytes.^[^ ^36^ ^]^ These immune mechanisms, however, were not addressed in this study, which could limit a comprehensive understanding of how microbiome‐based treatments like FMT might influence immune regulation in ARDS patients. This is an important area for future research, as alterations in the immune response through these pathways could be integral to the effects of FMT on ARDS pathogenesis.

**Figure 8:**
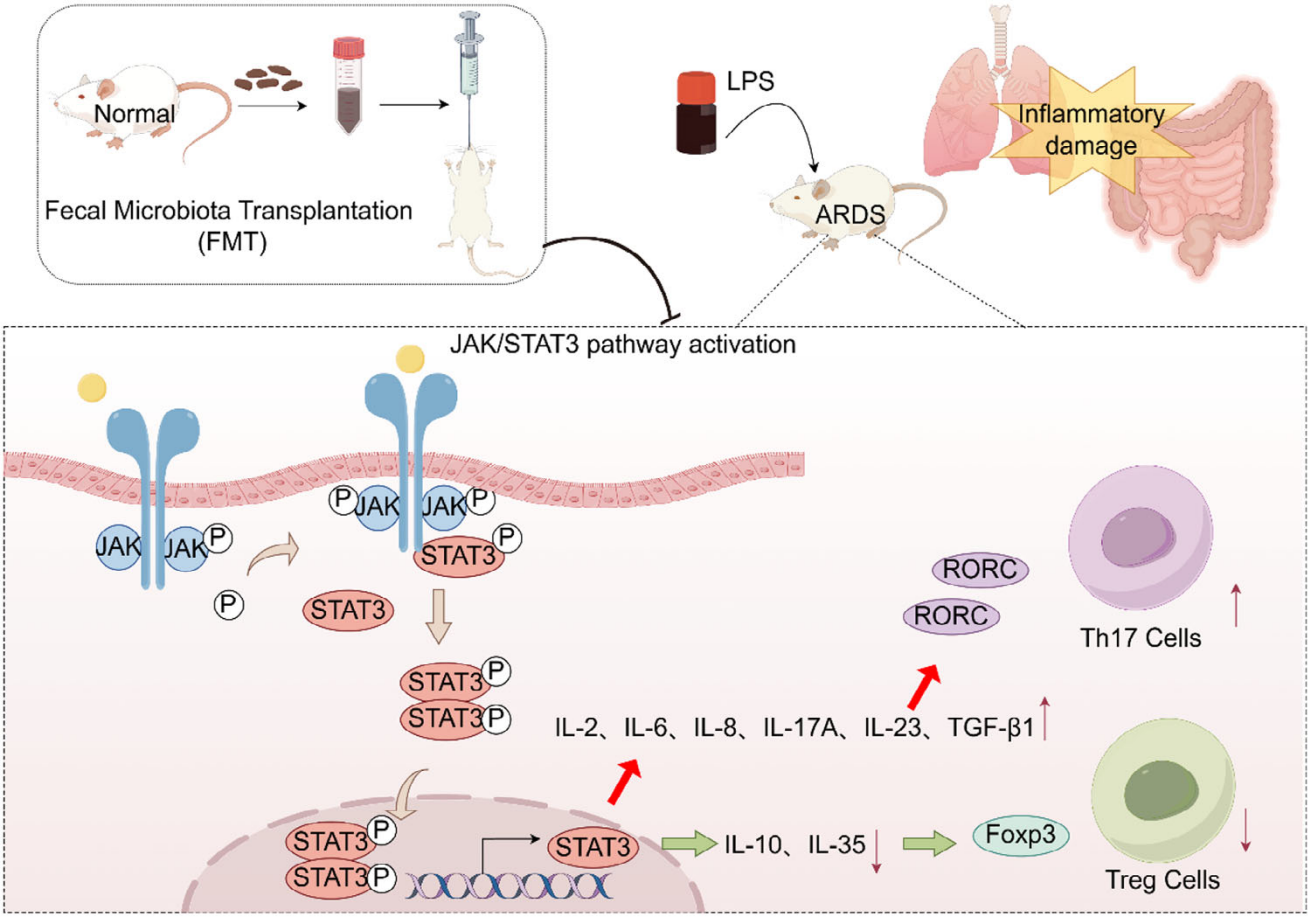
Mechanism of FMT modulating Th17/Treg balance via the JAK/STAT pathway.

In summary, this study demonstrates that FMT can inhibit the activity of the JAK/STAT signaling pathway, subsequently affecting the expression levels of RORγt and Foxp3, and restoring the balance of Th17/Treg cells. This effectively alleviates lung and intestinal damage in the LPS‐induced ARDS rat model (**Figure** **8**). These findings provide new experimental evidence for the potential application of FMT in the treatment of ARDS and offer important theoretical support for further exploration of the mechanisms of gut microbiota and immune regulation in ARDS.

## Conflict of Interest

The authors declare no conflict of interest.

## Author Contributions

D.Z. contributed to the formal analysis, conceptualization, supervision, visualization, and the original draft writing of the manuscript. B.D. was responsible for methodology, data curation, investigation, and conceptualization, and also participated in writing the original draft. J.C. contributed to the investigation, formal analysis, visualization, and original draft writing. Z.Z. was involved in data curation, software development, validation, and original draft writing. W.Z. contributed to methodology, formal analysis, investigation, and writing—review and editing. L.L. provided project administration, resources, validation, and contributed to writing—review, and editing. X.X. participated in software development, methodology, data curation, and writing—review, and editing. X.Q. contributed resources, project administration, funding acquisition, and writing—review, and editing. X.F. was involved in project administration, funding acquisition, and original draft writing.

## Data Availability

The data that support the findings of this study are available from the corresponding author upon reasonable request.
